# Biopolymer Composites 2022

**DOI:** 10.3390/ijms24076430

**Published:** 2023-03-29

**Authors:** Ana M. Díez-Pascual

**Affiliations:** Universidad de Alcalá, Facultad de Ciencias, Departamento de Química Analítica, Química Física e Ingeniería Química, Ctra. Madrid-Barcelona Km. 33.6, 28805 Alcalá de Henares, Madrid, Spain; am.diez@uah.es

Recently, sustainable, biodegradable, and nontoxic materials, especially from renewable resources, have gained a lot of attention, and an important effort has been put into the research of biodegradable and biocompatible polymers as an alternative to petroleum-based commodity plastics. However, biopolymers often show poor mechanical properties and limited processing capabilities and end-use applications. In order to overcome these disadvantages and build up advanced materials for a wide range of applications, biopolymers can be reinforced with fillers or nanofillers. The nanofillers possess a larger specific surface area and aspect ratio compared to conventional microfillers and result in materials that have novel and enhanced properties due to synergetic effects. Consequently, bionanocomposites are beneficial for numerous applications, from medicine and pharmaceutics to cosmetics and food packaging, to mention but a few of a long list. This Special Issue, with a collection of fifteen research articles and two reviews, provides select cases of the latest advances in the synthesis, characterization, and applications of sustainable and biodegradable biopolymer composites.

One of the most widely investigated is the well-known polylactic acid (PLA): an aliphatic polyester that can be produced from agricultural resources such as corn and through ring-opening polymerization of the lactides [1]. In this context, biocomposites comprising PLA-based matrices are regarded as very interesting due to their combination of good mechanical and physical properties and high sustainability [2,3]. In this regard, a new strategy for preparing block copolymers of PLA based on the introduction of multiple “inifer” groups such as tetraphenylethane (TPE) has been recently reported [4], and the copolymer showed improved properties such as higher glass transition temperature. Natural biopolymers include cellulose, chitosan, starch, collagen, gelatin, hyaluronic acid, alginates, fibrin, and pectin, which are widely found in nature. These biopolymers have displayed many interesting properties, including biocompatibility, biodegradability, and antibacterial activity [5]. For instance, a biocompatible and low-cost electrolyte-based synaptic transistor has been developed on a paper substrate comprising potato-starch electrolyte gate insulators and indium–gallium–zinc oxide (IGZO) channels [6]. This transistor can effectively mimic biological synapses; hence, it is expected to be a building block for bio-friendly neuromorphic computing systems in the future. Additionally, the structure, rheological properties, and film performance of wheat flour hydrocolloids have been accomplished and compared with those of wheat starch–gluten mixtures [7]. The incorporation of gluten reduces the inter-chain hydrogen bonding of starch, thus dropping its viscosity and solid-like behaviour while reducing the surface smoothness, compacity, water vapor barrier performance, and mechanical properties of the films. However, good compatibility between starch and gluten has been observed, which could improve the processability of the film’s blends. This work paves the way for the development of other flour-based edible packaging materials, thereby boosting energy conservation and eco-friendly protection.

On the other hand, a Pickering emulsion was developed with zein and sodium alginate polysaccharide as surface-active colloids to encapsulate hydrophobic Asta extract derived from Penaeus sinensis by-products. The properties of the resulting emulsion were measured, such as stability under heating, a different pH, and the treatment of metal ions and their degradation kinetics [8]. It shows high potential as an antioxidant additive in mild heating foods.

Carboxycellulose nanofibers (CNFs) also promise to be a green and inexpensive alternative material for polymer electrolyte membranes compared to the costly commercial Nafion. Nevertheless, its practical applications have been restricted by its relatively low performance and poor mechanical properties under standard operating conditions. In a recent study, CNFs were obtained from wood pulp by the TEMPO oxidation of the hydroxyl group that was present on the C6 position of the cellulose chain. Then, citric acid cross-linked CNF membranes were prepared by means of a solvent-casting method to improve their performance. The results from spectroscopic and diffraction techniques demonstrated the chemical cross-link between the citric acid and CNF, and the optimal fuel cell performance was assessed [9].

Chitosan, a natural polysaccharide, has also been widely investigated as a biomaterial, especially as a nanocarrier for drug delivery [10], as a scaffold [11], as an antibacterial wound dressing [12] or even for food packaging [13]. In particular, the potential of chitosan and carboxymethyl chitosan (CMC) cryogels cross-linked with diglycidyl ether of 1,4-butandiol (BDDGE) and poly(ethylene glycol) (PEGDGE) have been compared in terms of preventing the bacterial colonization of scaffolds. CMC cryogels were more efficient in preventing the adhesion and colonization of both *P. fluorescence* and *S. aureus*, demonstrating improved antifouling properties, while only moderate antimicrobial properties were observed [14]. In this regard, theoretical models on thermoreversible gelation are of great interest for predicting the properties of this type of cryogel [15]. In addition, the antimicrobial properties of chitosan, poly (vinyl alcohol), and silver nanoparticles were investigated. The ternary biocomposites are biocompatibles and were found to have antimicrobial activity against *S. aureus* [16]. Additionally, novel chitosan/tannic acid and chitosan/oxidized tannic acid composite films with excellent mechanical and antibacterial properties were made using a tape-casting method [17]. The results showed that when 20% tannic acid was added, the tensile strength of chitosan increased by about 90%, and could be attributed to the formation of a dual network structure comprising both chemical and hydrogen bonds that enhanced the mechanical properties. Moreover, these mixtures showed important antibacterial effects against *E. coli* bacteria and could be sued in food packaging applications. Other antimicrobial agents, such as antimicrobial peptides, have been recently investigated, which induce changes in membrane permeability and apoptosis [18].

Polysiloxanes have also gained a lot of interest in biomedical applications owing to their intrinsic properties, involving good flexibility and biocompatibility. Nevertheless, their poor mechanical strength limits their applications. A series of thermoplastic polysiloxane-polyurethanes were synthesized using hydroxyl-terminated polydimethylsiloxane, 4,4′-dicyclohexylmethane diisocyanate (HMDI), and 1,4-butanediol as raw materials. The resulting materials showed microphase separation, outstanding mechanical properties, and adequate processability. Their tensile strength was significantly higher than that of other flexible polysiloxane materials. Moreover, the tensile strength and breaking elongation were maintained after three cycles of regeneration. These novel biomaterials show great potential in applications such as gas separation, medical materials, antifouling coatings, and so forth [19]. Sustainable renewable polymer foams are a novel strategy that can effectively solve the problems of large surface density, high additive amount, and a narrow absorbing band of absorbing materials. In a recent study, novel renewable microwave-absorbing foams were prepared using kernel oil-based polyurethane foam as a porous matrix and Fe_3_O_4_-nanoparticles as magnetic absorbents [20]. The microstructure and microwave absorption performance, the structural effects on the properties, and the electromagnetic mechanism of the resulting materials were characterized and analysed in detail. The results suggest that this novel foam shows great promise in the field of lightweight stealth materials.

Damping materials, which can convert mechanical energy into thermal energy, can alleviate the problems caused by vibration and noise. Nevertheless, these materials are primarily based on petroleum-based resources, and their glass transition temperatures are lower than room temperature. Therefore, bio-based materials with high damping properties at room temperature are urgently required for green development. In this regard, a bio-based millable polyurethane with good high damping properties at room temperature has been developed [21] from modified PLA-based polyols. This study suggests a novel approach for designing materials that have high damping properties from sustainable resources.

On the other hand, water pollution by dyes represents a critical environmental problem; it is essential to produce new, effective, cost-attractive decolorization methods that can be used at the industrial level. Magnetic cyclodextrin polymers offer the advantage of easy separation from the dye solution. In a recent work [22], the β-cyclodextrin-epichlorohydrin polymer was synthesized, characterized, and tested for the removal of the azo dye Direct Red 83:1 from the water. It exhibited good adsorption properties and separability from water and allowed for the reuse of both the polymer and the dye in the dyeing process.

Another widely used biopolymer is polycaprolactone (PCL): a non-toxic, semicrystalline polyester with good processability and blend compatibility that is widely used in environmental and biomedical applications. However, PCL is expensive and has long biodegradability cycles; hence, it has been blended with cheaper biodegradable polymers such as polysaccharides, including starch, cellulose, chitin, and chitosan [23]. Recently, PCL membranes, which were produced by the electrospinning technique and incorporating gold complexes, have been prepared to evaluate their efficiency against melanoma cells and, therefore, their feasibility for use as an antitumoral patch for topical use [24].

Composites based on natural rubber also exhibit a lot of interest. In this regard, rubber reinforced with minerals (silica and chalk), sawdust, and hemp fillers have been prepared by peroxide cross-linking in the presence of trimethylolpropane trimethacrylate and irradiated by electron beam [25]. Their mechanical characteristics, gel fraction, cross-linking degree, water uptake, and weight loss in water and toluene were evaluated. Upon increasing the irradiation dose, the crosslinking and gel fraction decreased, hence indicating its mechanical properties.

Polymethyl methacrylate (PMMA) is the most well-known polymer of the methacrylate family. It is a low-dense, stable, and durable polymer that presents excellent biocompatibility and hemocompatibility, and high transparency, making it suitable for a wide variety of biomedical applications that require lasting, mechanically stable structures such as orthopaedics and bone tissue engineering [26]. In addition, its aesthetics, inexpensiveness, processability, versatility in terms of shaping, simple manipulation, low density, and adjustable mechanical properties make it a perfect candidate in the field of dentistry, such as the manufacture of artificial teeth, interim-fixed restorations, dentures, and denture bases, and so forth. However, PMMA lacks antimicrobial properties, shows high water absorption, and displays poor flexural and impact strength. With the aim of solving these issues, composites comprising natural or synthetic fibres or nanoparticles have been developed.
